# Reply to: Neurological outcome after cardiac arrest: cold and dark issues [editorial]

**DOI:** 10.5935/0103-507X.20160040

**Published:** 2016

**Authors:** Cristina Granja, Antonio Paulo Nassar Junior

**Affiliations:** 1 Department of Biomedical Sciences and Medicine, Universidade do Algarve - Faro, Portugal.; 2 Discipline of Clinical Emergency, Hospital das Clínicas, Faculdade de Medicina, Universidade de São Paulo - São Paulo (SP), Brazil; Adult Intensive Care Unit, A. C. Camargo Cancer Center - São Paulo (SP), Brazil.

We are thankful for the interest in our editorial.^(1)^ We agree that the study population in Kim et al.^(2)^ is different from that of Leão et al.^(3)^, insofar as the first consisted of patients who had pre-hospital cardiorespiratory arrest, and the second consisted of patients with out-of-hospital and in-hospital cardiorespiratory arrest. However, neither study showed benefits to achieving the target hypothermia more quickly. In addition, the study by Leão et al. suggested a worse prognosis in patients who reached hypothermia more quickly.^(3)^ As mentioned in the editorial, although the study had several limitations, there is a pathophysiological rationale for this finding.^(4,5)^

Obviously, we agree that the study of Leão et al.^(3)^ did not aim to assess the impact of temperature control. However, by showing that reaching hypothermia early was associated with worse neurological outcomes, the study adds to other recent evidence questioning the use of this therapeutic strategy.^(2,6)^ It is important to highlight that the two major studies in which the recommendation to apply hypothermia after spontaneous circulation is restored compared hypothermia with no intervention on the patients' temperature.^(7,8)^ In both studies, the control group had a core temperature above 37.5°C in the first 24 hours after recovery of spontaneous circulation. An important issue associated with these studies is that it is known that early hyperthermia after restoration of spontaneous circulation is associated with worse prognosis.^(9)^ Thus, the better outcomes associated with hypothermia may merely be a consequence of temperature control and not of the hypothermia itself in the intervention groups. The study by Nielsen et al. shows that this assumption may be true, as normothermia (36°C) led to results similar to hypothermia (33°C) in regards to mortality, neurological deficits^(6)^, and quality of life.^(10)^ Moreover, one of the studies mentioned above should be considered "quasi-randomized" considering the methodology employed.^(8)^

In addition to the evidence provided in the study by Nielsen et al.^(6)^, which showed no superiority of hypothermia in relation to normothermia, we believe that there are two obstacles for the indiscriminate adoption of hypothermia after cardiorespiratory arrest. First, the induction of hypothermia with infusion of cold saline solution, perhaps the most widely available method in our setting, is associated with increased risk of pulmonary edema,^(2)^ which could be a serious problem for patients with heart or kidney failure. Second, hypothermia is not exempt from complications, which include electrolyte disorders (hypokalemia, hypomagnesemia, and hypophosphatemia)^(11)^ and increased risk of infection.^(7,11,12)^ Based on these assumptions, we believe (for the time being and until further evidence is provided) that a core temperature of 36°C is a more viable option in our setting.

Cristina Granja

Department of Biomedical Sciences and Medicine, Universidade do Algarve - Faro, Portugal.

Antonio Paulo Nassar Junior

Discipline of Clinical Emergency, Hospital das Clínicas, Faculdade de Medicina, Universidade de São Paulo - São Paulo (SP), Brazil;

Adult Intensive Care Unit, A. C. Camargo Cancer Center - São Paulo (SP), Brazil.
